# 2-Meth­oxy-4-(prop-2-en-1-yl)phenyl 4-meth­oxy­benzoate

**DOI:** 10.1107/S1600536813011458

**Published:** 2013-05-04

**Authors:** Mallikarjuna Rao Pichika, Beng Kang Yew, Seik Weng Ng

**Affiliations:** a Department of Pharmaceutical Chemistry, International Medical University, 126 Jalan Bukit Jalil, 57000 Kuala Lumpur, Malaysia; b Department of Chemistry, University of Malaya, 50603 Kuala Lumpur, Malaysia; c Chemistry Department, Faculty of Science, King Abdulaziz University, PO Box 80203 Jeddah, Saudi Arabia

## Abstract

In the title compound, C_18_H_18_O_4_, the planes of the benzene rings are twisted by 81.60 (5)°. In the crystal, weak C—H⋯O hydrogen bonds link the mol­ecules into supra­molecular chains extending along the *a* axis.

## Related literature

For the structure of phenyl benzoate, see: Shibakami & Sekiya (1995 ▶).
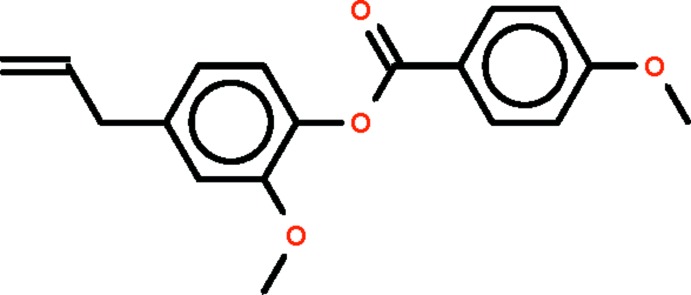


## Experimental

### 

#### Crystal data

C_18_H_18_O_4_
*M* *_r_* = 298.32
Triclinic, 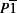

*a* = 8.7685 (6) Å
*b* = 9.8159 (7) Å
*c* = 10.3515 (6) Å
α = 113.030 (6)°
β = 101.231 (6)°
γ = 102.378 (6)°
*V* = 761.45 (11) Å^3^
*Z* = 2
Mo *K*α radiation
μ = 0.09 mm^−1^
*T* = 100 K
0.40 × 0.40 × 0.20 mm

#### Data collection

Agilent SuperNova Dual diffractometer with an Atlas detector
Absorption correction: multi-scan (*CrysAlis PRO*; Agilent, 2013 ▶) *T*_min_ = 0.964, *T*_max_ = 0.982
6267 measured reflections
3525 independent reflections
2497 reflections with *I* > 2σ(*I*)
*R*_int_ = 0.027

#### Refinement

*R*[*F* ^2^ > 2σ(*F* ^2^)] = 0.050
*wR*(*F* ^2^) = 0.132
*S* = 1.06
3525 reflections
199 parameters
H-atom parameters constrained
Δρ_max_ = 0.47 e Å^−3^
Δρ_min_ = −0.24 e Å^−3^

### 

Data collection: *CrysAlis PRO* (Agilent, 2013 ▶); cell refinement: *CrysAlis PRO*; data reduction: *CrysAlis PRO*; program(s) used to solve structure: *SHELXS97* (Sheldrick, 2008 ▶); program(s) used to refine structure: *SHELXL97* (Sheldrick, 2008 ▶); molecular graphics: *X-SEED* (Barbour, 2001 ▶); software used to prepare material for publication: *publCIF* (Westrip, 2010 ▶).

## Supplementary Material

Crystal structure: contains datablock(s) global, I. DOI: 10.1107/S1600536813011458/xu5699sup1.cif

Structure factors: contains datablock(s) I. DOI: 10.1107/S1600536813011458/xu5699Isup2.hkl

Supplementary material file. DOI: 10.1107/S1600536813011458/xu5699Isup3.cml

Additional supplementary materials:  crystallographic information; 3D view; checkCIF report

## Figures and Tables

**Table 1:** Hydrogen-bond geometry (Å, °)

*D*—H⋯*A*	*D*—H	H⋯*A*	*D*⋯*A*	*D*—H⋯*A*
C18—H18*B*⋯O3^i^	0.98	2.54	3.458 (2)	156

Symmetry code: (i) 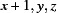.

**Fig. 1.**
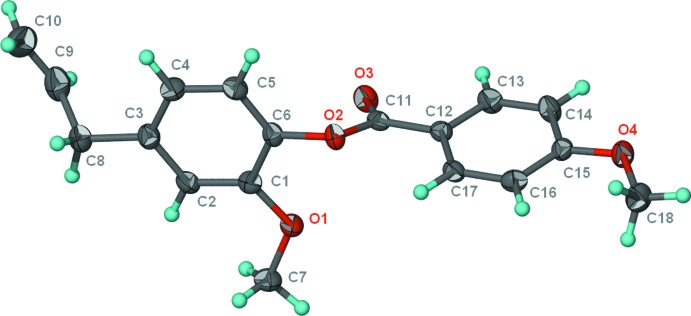
Thermal ellipsoid plot (Barbour, 2001) of C18H18O4 at the 70% probability level; hydrogen atoms are drawn as spheres of arbitrary radius.

## supplementary crystallographic information

### Comment

The title phenyl benzoate (Scheme I, Fig. 1), which possesses an allyl and a methoxy substituent, was synthesized for an evaluation of its pharmaceutical properties as it is an ester derivative of eugenol. The two benzene rings are approximately perpendicular [dihedral angle 81.60 (5)°]. The twist is similar to that found in the unsubstituted compound, phenyl benzoate (Shibakami & Sekiya, 1995). In the crystal, weak C—H···O hydrogen bond links molecules into the supramolecular chains extending along the a axis (Table 1).

### Experimental

4-Allyl-2-methoxyphenol (1 mmol), 4-methoxybenzoic acid (1 mmol), diethylazodicarboxylate (2 mmol) and triphenylphosphine (2 mmol) were heated in THF (10 ml) for 2 h. The solid material extracted with dichloromethane. The dichloromethane solution was eluted through a silica gel column by using an *n*-hexane–ethyl acetate (95: 5 *v*/*v*) solvent system. Slow evaporation of the solution yielded large colorless crystals.

### Refinement

Carbon-bound H-atoms were placed in calculated positions [C–H 0.95 to 0.98 Å, *U*_iso_(H) 1.2 to 1.5*U*_eq_(C)] and were included in the refinement in the riding model approximation.

### Crystal data

**Table:**

C_18_H_18_O_4_	*Z* = 2
*M**_r_* = 298.32	*F*(000) = 316
Triclinic, *P*1	*D*_x_ = 1.301 Mg m^−^^3^
Hall symbol: -P 1	Mo *K*α radiation, λ = 0.71073 Å
*a* = 8.7685 (6) Å	Cell parameters from 2058 reflections
*b* = 9.8159 (7) Å	θ = 3.4–27.5°
*c* = 10.3515 (6) Å	µ = 0.09 mm^−^^1^
α = 113.030 (6)°	*T* = 100 K
β = 101.231 (6)°	Prism, colorless
γ = 102.378 (6)°	0.40 × 0.40 × 0.20 mm
*V* = 761.45 (11) Å^3^	

### Data collection

**Table:**

Agilent SuperNova Dual diffractometer with an Atlas detector	3525 independent reflections
Radiation source: SuperNova (Mo) X-ray Source	2497 reflections with *I* > 2σ(*I*)
Mirror monochromator	*R*_int_ = 0.027
Detector resolution: 10.4041 pixels mm^-1^	θ_max_ = 27.6°, θ_min_ = 3.4°
ω scan	*h* = −11→8
Absorption correction: multi-scan (*CrysAlis PRO*; Agilent, 2013)	*k* = −11→12
*T*_min_ = 0.964, *T*_max_ = 0.982	*l* = −13→13
6267 measured reflections	

### Refinement

**Table:**

Refinement on *F*^2^	Primary atom site location: structure-invariant direct methods
Least-squares matrix: full	Secondary atom site location: difference Fourier map
*R*[*F*^2^ > 2σ(*F*^2^)] = 0.050	Hydrogen site location: inferred from neighbouring sites
*wR*(*F*^2^) = 0.132	H-atom parameters constrained
*S* = 1.06	*w* = 1/[σ^2^(*F*_o_^2^) + (0.0533*P*)^2^ + 0.0956*P*] where *P* = (*F*_o_^2^ + 2*F*_c_^2^)/3
3525 reflections	(Δ/σ)_max_ = 0.001
199 parameters	Δρ_max_ = 0.47 e Å^−^^3^
0 restraints	Δρ_min_ = −0.24 e Å^−^^3^

### Fractional atomic coordinates and isotropic or equivalent isotropic displacement parameters (Å^2^)

**Table:**

	*x*	*y*	*z*	*U*_iso_*/*U*_eq_	
O1	0.25854 (15)	0.56195 (13)	0.97044 (12)	0.0239 (3)	
O2	0.17691 (14)	0.49329 (12)	0.68408 (12)	0.0201 (3)	
O3	0.03197 (15)	0.26310 (13)	0.66616 (13)	0.0242 (3)	
O4	0.54829 (15)	0.06265 (13)	0.33989 (13)	0.0245 (3)	
C1	0.1367 (2)	0.61293 (18)	0.92258 (18)	0.0198 (4)	
C2	0.0589 (2)	0.70167 (19)	1.01000 (18)	0.0212 (4)	
H2	0.0890	0.7300	1.1126	0.025*	
C3	−0.0628 (2)	0.75000 (19)	0.94958 (18)	0.0223 (4)	
C4	−0.1092 (2)	0.70584 (19)	0.79870 (18)	0.0228 (4)	
H4	−0.1929	0.7373	0.7565	0.027*	
C5	−0.0329 (2)	0.61580 (19)	0.70993 (18)	0.0208 (4)	
H5	−0.0646	0.5852	0.6069	0.025*	
C6	0.0886 (2)	0.57120 (18)	0.77169 (17)	0.0186 (4)	
C7	0.3292 (2)	0.6251 (2)	1.12758 (18)	0.0273 (4)	
H7A	0.4173	0.5827	1.1487	0.041*	
H7B	0.2443	0.5963	1.1700	0.041*	
H7C	0.3742	0.7392	1.1712	0.041*	
C8	−0.1423 (2)	0.8524 (2)	1.0485 (2)	0.0285 (4)	
H8A	−0.0931	0.8723	1.1514	0.034*	
H8B	−0.1166	0.9544	1.0460	0.034*	
C9	−0.3225 (2)	0.7840 (2)	1.0064 (2)	0.0342 (5)	
H9	−0.3629	0.6969	1.0236	0.041*	
C10	−0.4308 (3)	0.8330 (3)	0.9476 (2)	0.0412 (5)	
H10A	−0.3957	0.9198	0.9284	0.049*	
H10B	−0.5447	0.7819	0.9239	0.049*	
C11	0.1434 (2)	0.33704 (18)	0.64360 (17)	0.0181 (4)	
C12	0.2555 (2)	0.27352 (18)	0.56621 (17)	0.0179 (4)	
C13	0.2089 (2)	0.11166 (19)	0.47938 (18)	0.0232 (4)	
H13	0.1070	0.0463	0.4717	0.028*	
C14	0.3087 (2)	0.04630 (19)	0.40522 (19)	0.0241 (4)	
H14	0.2745	−0.0635	0.3452	0.029*	
C15	0.4596 (2)	0.14002 (19)	0.41746 (17)	0.0195 (4)	
C16	0.5092 (2)	0.30149 (19)	0.50483 (18)	0.0210 (4)	
H16	0.6126	0.3663	0.5146	0.025*	
C17	0.4059 (2)	0.36649 (18)	0.57741 (17)	0.0193 (4)	
H17	0.4387	0.4765	0.6357	0.023*	
C18	0.7088 (2)	0.1525 (2)	0.3554 (2)	0.0301 (4)	
H18A	0.7575	0.0844	0.2908	0.045*	
H18B	0.7782	0.1986	0.4582	0.045*	
H18C	0.7004	0.2360	0.3277	0.045*	

### Atomic displacement parameters (Å^2^)

**Table:**

	*U*^11^	*U*^22^	*U*^33^	*U*^12^	*U*^13^	*U*^23^
O1	0.0262 (7)	0.0270 (6)	0.0171 (6)	0.0127 (5)	0.0042 (5)	0.0077 (5)
O2	0.0243 (6)	0.0178 (6)	0.0196 (6)	0.0080 (5)	0.0107 (5)	0.0073 (5)
O3	0.0241 (7)	0.0222 (6)	0.0247 (7)	0.0046 (5)	0.0117 (5)	0.0085 (5)
O4	0.0221 (6)	0.0247 (6)	0.0262 (7)	0.0084 (5)	0.0119 (5)	0.0084 (5)
C1	0.0212 (9)	0.0180 (8)	0.0202 (9)	0.0055 (7)	0.0055 (7)	0.0096 (7)
C2	0.0226 (9)	0.0209 (8)	0.0157 (8)	0.0050 (7)	0.0050 (7)	0.0055 (7)
C3	0.0213 (9)	0.0214 (8)	0.0228 (9)	0.0062 (7)	0.0103 (7)	0.0075 (7)
C4	0.0223 (9)	0.0245 (9)	0.0225 (9)	0.0085 (7)	0.0054 (8)	0.0118 (7)
C5	0.0229 (9)	0.0227 (8)	0.0168 (8)	0.0057 (7)	0.0065 (7)	0.0095 (7)
C6	0.0205 (8)	0.0167 (8)	0.0188 (8)	0.0054 (7)	0.0091 (7)	0.0069 (7)
C7	0.0290 (10)	0.0306 (10)	0.0190 (9)	0.0110 (8)	0.0017 (8)	0.0098 (8)
C8	0.0293 (10)	0.0308 (10)	0.0227 (9)	0.0136 (8)	0.0096 (8)	0.0067 (8)
C9	0.0378 (12)	0.0304 (10)	0.0362 (11)	0.0124 (9)	0.0209 (10)	0.0114 (9)
C10	0.0325 (11)	0.0520 (13)	0.0377 (12)	0.0177 (10)	0.0126 (10)	0.0161 (10)
C11	0.0198 (8)	0.0185 (8)	0.0133 (8)	0.0050 (7)	0.0026 (7)	0.0062 (6)
C12	0.0182 (8)	0.0192 (8)	0.0152 (8)	0.0052 (7)	0.0047 (7)	0.0072 (6)
C13	0.0216 (9)	0.0206 (8)	0.0240 (9)	0.0024 (7)	0.0086 (8)	0.0084 (7)
C14	0.0266 (9)	0.0171 (8)	0.0244 (9)	0.0047 (7)	0.0103 (8)	0.0052 (7)
C15	0.0204 (8)	0.0229 (8)	0.0174 (8)	0.0102 (7)	0.0065 (7)	0.0094 (7)
C16	0.0178 (8)	0.0208 (8)	0.0226 (9)	0.0021 (7)	0.0045 (7)	0.0110 (7)
C17	0.0210 (9)	0.0165 (8)	0.0173 (8)	0.0052 (7)	0.0041 (7)	0.0060 (7)
C18	0.0219 (9)	0.0332 (10)	0.0345 (11)	0.0083 (8)	0.0148 (8)	0.0119 (8)

### Geometric parameters (Å, º)

**Table:**

O1—C1	1.362 (2)	C8—H8A	0.9900
O1—C7	1.4381 (19)	C8—H8B	0.9900
O2—C11	1.3676 (19)	C9—C10	1.306 (3)
O2—C6	1.4110 (19)	C9—H9	0.9500
O3—C11	1.205 (2)	C10—H10A	0.9500
O4—C15	1.358 (2)	C10—H10B	0.9500
O4—C18	1.433 (2)	C11—C12	1.475 (2)
C1—C2	1.386 (2)	C12—C17	1.388 (2)
C1—C6	1.398 (2)	C12—C13	1.400 (2)
C2—C3	1.395 (2)	C13—C14	1.372 (2)
C2—H2	0.9500	C13—H13	0.9500
C3—C4	1.393 (2)	C14—C15	1.391 (2)
C3—C8	1.522 (2)	C14—H14	0.9500
C4—C5	1.389 (2)	C15—C16	1.396 (2)
C4—H4	0.9500	C16—C17	1.390 (2)
C5—C6	1.375 (2)	C16—H16	0.9500
C5—H5	0.9500	C17—H17	0.9500
C7—H7A	0.9800	C18—H18A	0.9800
C7—H7B	0.9800	C18—H18B	0.9800
C7—H7C	0.9800	C18—H18C	0.9800
C8—C9	1.478 (3)		
			
C1—O1—C7	116.79 (13)	C10—C9—C8	125.6 (2)
C11—O2—C6	116.91 (13)	C10—C9—H9	117.2
C15—O4—C18	117.48 (13)	C8—C9—H9	117.2
O1—C1—C2	125.77 (15)	C9—C10—H10A	120.0
O1—C1—C6	115.87 (14)	C9—C10—H10B	120.0
C2—C1—C6	118.36 (15)	H10A—C10—H10B	120.0
C1—C2—C3	120.95 (16)	O3—C11—O2	122.70 (15)
C1—C2—H2	119.5	O3—C11—C12	125.80 (15)
C3—C2—H2	119.5	O2—C11—C12	111.46 (14)
C4—C3—C2	119.50 (16)	C17—C12—C13	118.63 (15)
C4—C3—C8	120.39 (16)	C17—C12—C11	123.04 (14)
C2—C3—C8	120.10 (15)	C13—C12—C11	118.32 (15)
C5—C4—C3	119.97 (16)	C14—C13—C12	120.75 (16)
C5—C4—H4	120.0	C14—C13—H13	119.6
C3—C4—H4	120.0	C12—C13—H13	119.6
C6—C5—C4	119.76 (15)	C13—C14—C15	120.34 (15)
C6—C5—H5	120.1	C13—C14—H14	119.8
C4—C5—H5	120.1	C15—C14—H14	119.8
C5—C6—C1	121.45 (15)	O4—C15—C14	115.22 (14)
C5—C6—O2	119.49 (14)	O4—C15—C16	125.00 (15)
C1—C6—O2	118.84 (14)	C14—C15—C16	119.78 (15)
O1—C7—H7A	109.5	C17—C16—C15	119.31 (15)
O1—C7—H7B	109.5	C17—C16—H16	120.3
H7A—C7—H7B	109.5	C15—C16—H16	120.3
O1—C7—H7C	109.5	C12—C17—C16	121.17 (15)
H7A—C7—H7C	109.5	C12—C17—H17	119.4
H7B—C7—H7C	109.5	C16—C17—H17	119.4
C9—C8—C3	114.01 (15)	O4—C18—H18A	109.5
C9—C8—H8A	108.8	O4—C18—H18B	109.5
C3—C8—H8A	108.7	H18A—C18—H18B	109.5
C9—C8—H8B	108.7	O4—C18—H18C	109.5
C3—C8—H8B	108.7	H18A—C18—H18C	109.5
H8A—C8—H8B	107.6	H18B—C18—H18C	109.5
			
C7—O1—C1—C2	−9.9 (2)	C3—C8—C9—C10	−109.0 (2)
C7—O1—C1—C6	169.69 (14)	C6—O2—C11—O3	8.1 (2)
O1—C1—C2—C3	178.83 (15)	C6—O2—C11—C12	−173.97 (12)
C6—C1—C2—C3	−0.8 (2)	O3—C11—C12—C17	−161.98 (17)
C1—C2—C3—C4	1.3 (3)	O2—C11—C12—C17	20.1 (2)
C1—C2—C3—C8	−177.75 (16)	O3—C11—C12—C13	17.0 (2)
C2—C3—C4—C5	−0.7 (3)	O2—C11—C12—C13	−160.88 (14)
C8—C3—C4—C5	178.28 (15)	C17—C12—C13—C14	−0.9 (3)
C3—C4—C5—C6	−0.3 (3)	C11—C12—C13—C14	−179.88 (15)
C4—C5—C6—C1	0.7 (3)	C12—C13—C14—C15	1.2 (3)
C4—C5—C6—O2	−173.71 (14)	C18—O4—C15—C14	−176.50 (15)
O1—C1—C6—C5	−179.87 (15)	C18—O4—C15—C16	3.6 (2)
C2—C1—C6—C5	−0.2 (2)	C13—C14—C15—O4	179.68 (16)
O1—C1—C6—O2	−5.4 (2)	C13—C14—C15—C16	−0.5 (3)
C2—C1—C6—O2	174.27 (14)	O4—C15—C16—C17	179.26 (15)
C11—O2—C6—C5	−107.13 (17)	C14—C15—C16—C17	−0.6 (2)
C11—O2—C6—C1	78.29 (18)	C13—C12—C17—C16	−0.2 (2)
C4—C3—C8—C9	59.1 (2)	C11—C12—C17—C16	178.76 (15)
C2—C3—C8—C9	−121.88 (19)	C15—C16—C17—C12	0.9 (2)

### Hydrogen-bond geometry (Å, º)

**Table:**

*D*—H···*A*	*D*—H	H···*A*	*D*···*A*	*D*—H···*A*
C18—H18*B*···O3^i^	0.98	2.54	3.458 (2)	156

Symmetry code: (i) *x*+1, *y*, *z*.
